# Implications of Coding Layers on Physical-Layer Security: A Secrecy Benefit Approach

**DOI:** 10.3390/e21080755

**Published:** 2019-08-01

**Authors:** Willie K. Harrison, Elise Beard, Scott Dye, Erin Holmes, Kaela Nelson, Marco A. C. Gomes, João P. Vilela

**Affiliations:** 1 Department of Electrical and Computer Engineering, Brigham Young University, Provo, UT 84602, USA; 2 Department of Mathematics and Computer Science, Colorado College, Colorado Springs, CO 80903, USA; 3 Instituto de Telecomunicações, Department of Electrical and Computer Engineering, University of Coimbra, 3004-531 Coimbra, Portugal; 4 CISUC and Department of Informatics Engineering, University of Coimbra, 3004-531 Coimbra, Portugal

**Keywords:** physical-layer security, equivocation, Gaussian wiretap channel, concatenated coding

## Abstract

In this work, we consider the pros and cons of using various layers of keyless coding to achieve secure and reliable communication over the Gaussian wiretap channel. We define a new approach to information theoretic security, called practical secrecy and the secrecy benefit, to be used over real-world channels and finite blocklength instantiations of coding layers, and use this new approach to show the fundamental reliability and security implications of several coding mechanisms that have traditionally been used for physical-layer security. We perform a systematic/structured analysis of the effect of error-control coding, scrambling, interleaving, and coset coding, as coding layers of a secrecy system. Using this new approach, scrambling and interleaving are shown to be of no effect in increasing information theoretic security, even when measuring the effect at the output of the eavesdropper’s decoder. Error control coding is shown to present a trade-off between secrecy and reliability that is dictated by the chosen code and the signal-to-noise ratios at the legitimate and eavesdropping receivers. Finally, the benefits of secrecy coding are highlighted, and it is shown how one can shape the secrecy benefit according to system specifications using combinations of different layers of coding to achieve both reliable and secure throughput.

## 1. Introduction

Physical-layer security [1] is currently undergoing a resurgence of interest and activity, specifically regarding progressing towards real-world application of information theoretic security principles [2,3,4]. Some of the early works in information theoretic security [5,6,7] laid the foundations of secrecy capacity for various versions of the wiretap channel. Secrecy coding constructions to date [8,9] are known explicitly, however, only for discrete memoryless versions of the wiretap channel, and, hence, appear to fall short of solving the practical security issues for real channels.

Since explicit secrecy code constructions that achieve information theoretic security over the Gaussian wiretap channel are still missing [10,11], non-information theoretic security metrics have arisen to address practical concerns, e.g., the security gap [12,13], degrees of freedom in decoders [14], etc. Although some of these security metrics allow for analysis over any wiretap channel model, they have met resistance due to their weaker security guarantees. These approaches are typically based on probability of error analysis at the eavesdropper assuming a specific decoder, rather than information theoretic analysis that would hold regardless of the chosen decoder. More recently, however, an additional information theoretic security approach has begun to take shape, where the eavesdropper’s decoder outputs are used to estimate the security of a system [10,15,16]. This allows one to precisely and efficiently conduct the security analysis of systems over any wiretap channel variation through Monte Carlo simulation [4]. Since this new technique must also choose a specific decoder for the eavesdropper, it therefore requires one to assume that the eavesdropper will use the best (and hopefully provably best) decoder available. In this paper, we formalize the new information theoretic security definition and say that a code *achieves practical secrecy* if the entropy of the message given the best known decoder output can be shown to be approximately equal to the entropy of the message at the eavesdropper. Since the metric is a practical one, its application to finite blocklength codes with known explicit constructions is highlighted herein. Along with this new security metric, we present the idea of a code’s *secrecy benefit*, which, over the Gaussian wiretap channel, shows the increase in confusion at the eavesdropper for the coded scenario over the uncoded scenario as a function of signal-to-noise ratio.

In addition to presenting these new metrics, we showcase their application in analyzing various coding approaches over the Gaussian wiretap channel. Although explicit code constructions exist that can both correct errors for legitimate parties and keep secrets from eavesdroppers over discrete memoryless wiretap channels [8,9,17], some works have sought solutions to tandem secrecy and reliability coding for real-world channels using a layered, or concatenated, coding approach [10,18,19]. The contributions of this paper include a systematic and structured analysis of error-control coding, interleaving, scrambling, and coset-based secrecy coding, as layers in a physical-layer security coding system. We chose these layers so as to represent fundamentally different approaches to coding for secrecy over the Gaussian wiretap channel, and leave additional layers of coding, such as other constructions of wiretap codes [8,9], for future work. Note also that scrambling and interleaving have been considered as keyed layers of coding as well [18,19], although herein we only consider keyless versions of these. The analysis of both reliability and security is carried out for each of the layers in isolation, as well as for various combinations of layers of coding. Benefits and drawbacks are provided for the inclusion of each layer in a concatenated coding system for physical-layer security. Among them, we show that keyless layers of scrambling and interleaving provide no secrecy benefit over the uncoded case, error-control coding layers can be used to increase security or reliability depending on the signal-to-noise ratios at the legitimate and eavesdropping receivers, and the secrecy benefit of coset-based secrecy codes can be shaped using error-control coding.

The rest of the paper is organized as follows. Section 2 provides the setup for the paper, including the system model and the metrics that define our new approach to information theoretic security. In Section 3, we consider layers of coding for reliable data transfer between legitimate nodes in a network, and show how error-control coding must leak information in the strictest sense of security, but, with the new approach, it may be considered a help to security efforts for a range of signal-to-noise ratios at the eavesdropper’s receiver. Layers of secure coding are considered in Section 4, where we start with simpler (and more controversial) layers of coding such as interleaving and scrambling, and move on to wiretap coding. Several combinations of layers of coding are then analyzed and discussed in Section 5, and conclusions are made in Section 6.

## 2. Setup

In this section, we establish the system model and discuss metrics for quantifying both reliability and security. For notation, we assign capital letters to random variables and matrices, lowercase letters to realizations of random variables, calligraphic letters to ranges of random variables, superscripts to indicate the size of vectors and matrices, and subscripts to index the elements of vectors and matrices.

### 2.1. System Model

**Figure 1:**
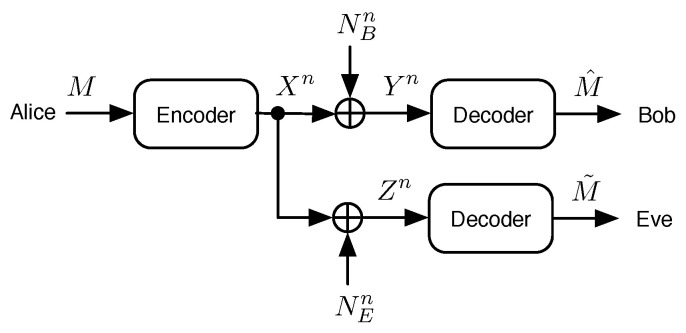
The Gaussian wiretap channel model.

In this paper, we consider the Gaussian wiretap channel model, as portrayed in Figure 1, where Alice wishes to send a message *M* chosen uniformly at random from $M=\{0,1,\ldots ,2^{k}-1\}$ to Bob in the presence of an eavesdropper named Eve. Alice encodes the message to produce a length-*n* binary codeword, which is modulated to produce a set of *n* source symbols $X^{n}$, which are then transmitted over two parallel independent Gaussian channels. The main channel of communications dictates Bob’s received signal, and we call the set of Bob’s decision variables $Y^{n}$, which are then decoded to produce Bob’s estimate of the message $\hat{M}$. Eve’s received signal is obtained over the eavesdropper’s channel, and the set of Eve’s decision variables are denoted $Z^{n}$. Eve may choose to attempt to decode the data as well. If she does, then her estimate of the message is called $\tilde{M}$. When considering $\tilde{M}$ in this paper, we assume that Eve uses the best known decoder to produce her estimate of the message. Additive white circularly symmetric zero-mean Gaussian noise processes at the main and eavesdropper’s channels are denoted $N_{B}^{n}$ and $N_{E}^{n}$, respectively, and thus,

(1) $$\begin{array}{cc} Y^{n} & =X^{n}+N_{B}^{n}, \end{array}$$

(2) $$\begin{array}{cc} Z^{n} & =X^{n}+N_{E}^{n}. \end{array}$$

The respective variances in all dimensions of the signal space for noise processes $N_{B}^{n}$ and $N_{E}^{n}$ are denoted $\sigma_{B}^{2}$ and $\sigma_{E}^{2}$.

**Figure 2:**
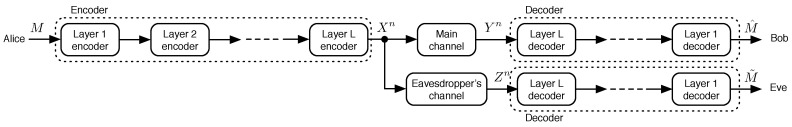
Portrayal of a layered, or concatenated, coding approach to physical-layer security.

The encoder and decoder may be comprised of several layers of coding, as shown in Figure 2. All codes are block codes. The dimension of each encoding layer is equal to the number of input bits required at the layer to do a single encoding operation, while the blocklength of each encoding layer is equal to the number of output bits produced by the layer in a single encoding operation. Unless otherwise stated, it is assumed that each symbol of the message *M* is encoded separately at each layer. That is, the dimension of the Layer *i* encoder matches exactly the blocklength of the Layer (i−1) encoder for i=2,3,…,L. Thus, there is no need to buffer codewords at any layer of the encoding or decoding process to perform the operations. For the Layer 1 encoder, a message symbol from *M* is first mapped to *k* bits, and *k* is the dimension of Layer 1. All codes are assumed to be binary, and coding operations are computed in $F_{2}$. The rate of each coding layer is simply given as the ratio of the layer’s dimension over its blocklength. The digital modulator and demodulator are assumed to be in place [20], but are not depicted as *layers* in Figure 2.

### 2.2. Metrics

The equivocation $H(M|Z^{n})$ is now a standard metric to consider when analyzing security over the wiretap channel model. If

(3) $$H\left(M|Z^{n}\right)=H\left(M\right),$$

we may say the system achieves *perfect secrecy* in some sense, although the original Shannon definition of perfect secrecy [21] required that $H\left(M|X^{n}\right)=H\left(M\right)$. If we can only state that for a sequence of codes with blocklength *n*

(4) $$\lim_{n\to \infty }\frac{1}{n}H\left(M|Z^{n}\right)=H\left(M\right),$$

then the system is said to achieve *weak secrecy*, while

(5) $$\lim_{n\to \infty }H\left(M|Z^{n}\right)=H\left(M\right)$$

indicates that a system achieves *strong secrecy*. Each of these security definitions assumes that messages are chosen uniformly at random from M. If the strong secrecy condition can be shown to hold for any possible distribution on the messages, then we may state that the system achieves *semantic secrecy*. Of note to this work is that it has been shown recently in [11] that lattice codes can be used to achieve semantic secrecy over the Gaussian wiretap channel, and yet knowledge of how to build such codes is still forthcoming. Assuming we could find constructions to achieve information theoretic security in any sense over the Gaussian wiretap channel, we also note that, other than for perfect secrecy, the security analysis requires blocklength to grow in the limit to infinity. It remains unclear as to how these security measures should or should not be changed for finite blocklength codes, although the problem has been addressed using various approaches [4,22,23,24].

While the general trend has been to employ stricter definitions of secrecy over time as codes have been discovered that achieve weak, then strong, then semantic secrecy, albeit only for discrete memoryless wiretap channel model variants [8,9], in this paper, we move in the opposite direction in order to formulate a security metric that has immediate application over real-world channels when finite blocklength codes are deployed. The new metric is more practical than many of the traditional metrics, and is to be computed for specific codes of specific blocklengths. The metric also returns a feel for the operational level of security, seeing as any real system will require the eavesdropper to attempt to decode the message. Some suggest that security metrics based on bit-error rate (BER) may be of use [12,13] when information theoretical metrics prove difficult to use, since in practice we really only care if Eve can decode and obtain the secret message. However, it is still desirable to root even practical metrics in information theory, even if we must analyze Eve’s decoder outputs rather than $Z^{n}$.

**Definition** **1.** *We say that a finite blocklength coding system achieves* practical secrecy *at a level of $\delta_{s}$ if, for the best known decoding algorithm,*

(6) $$H\left(M|\tilde{M}\right)-H\left(M\right)<\delta_{s}.$$

In other words, we’d like $H\left(M|\tilde{M}\right)\approx H\left(M\right)$. This notion of secrecy has been used over the Gaussian wiretap channel in a few previous works [10,15,16]. One of the main reasons this notion of secrecy is so attractive is that, when Eve is forced to decode, a continuous random variable $Z^{n}$ is transformed into a discrete random variable $\tilde{M}$, which makes the entropy calculation straightforward. Note that, when the code’s error properties are not a function of the choice of m∈M, then

(7)H(M|M˜)=−∑m˜∈M∑m∈MpM,M˜(m,m˜)log2pM|M˜(m|m˜)(8)=−∑m∈MpM(m)∑m˜∈MpM˜|M(m˜|m)log2pM˜|M(m˜|m)pM(m)pM˜(m˜)(9)=−1|M|∑m∈M∑m˜∈MpM˜|M(m˜|m)log2pM˜|M(m˜|m)(10)=−∑m˜∈MpM˜|M(m˜|m)log2pM˜|M(m˜|m),

for any choice of m∈M. If |M| is small enough, this quantity can be estimated through Monte Carlo simulation of the encoding and decoding layers [4,10].

The goals of communication over the Gaussian wiretap channel for this paper are then to analyze and test specific layers of coding to ascertain whether they can be used to achieve both of the following constraints in a layered coding architecture: (i) $\mathrm{Pr}\left(M\neq \hat{M}\right)<\delta_{r}$ (the reliability constraint), and (ii) $H\left(M|\tilde{M}\right)-H\left(M\right)<\delta_{s}$ (the practical secrecy constraint), for positive $\delta_{r}$ and $\delta_{s}$ as small as possible and fixed *n*.

We will find it useful to compare several various coded cases with the uncoded case in terms of reliability and secrecy. A new mechanism for showcasing the usefulness of a coding technique for physical-layer security can help.

**Definition** **2.** *Let $H_{U}\left(M|\tilde{M}\right)$ be the practical secrecy when no coding layers are employed in the layered coding system of Figure 2, and let $H_{C}\left(M|\tilde{M}\right)$ be the practical secrecy measure when code C is employed in the system, where C may comprise one or more layers of coding. Then the* secrecy benefit *of C is defined as*

(11) $$B\left(C\right)=H_{C}\left(M|\tilde{M}\right)-H_{U}\left(M|\tilde{M}\right),$$

*which measures the uncertainty about M added at a receiving node over and above the uncertainty level of uncoded transmissions.*

We will find it useful to plot the secrecy benefit of many codes as a function of the signal-to-noise ratio in the eavesdropper’s channel.

## 3. Layers of Coding for Reliable Data Transfer

In this section, we discuss the general effects of adding a layer of error-control coding at Layer *L* in Figure 2. If the binary input to this encoder is *k* bits and the binary output is *n* bits, then we say the rate of the code is R=k/n. The overhead of the code is used to detect and correct errors [25]. Intuitively, we may decide to add a code of this nature to fine tune the reliability measure for Bob over the main channel, with the goal to retain as much security against Eve as possible. This approach has been highlighted in the recent works [24,26], where the eavesdropper’s channel state is assumed to be unknown. Rather than worry about achieving information theoretic security against Eve, one may instead decide to use the best existing code for secrecy to maximize the equivocation subject to the reliability constraint. In this way, coding for secrecy becomes an optimization problem rather than a strict security problem, and security endeavors at other layers in the protocol stack, e.g., cryptography, fill in to help when needed.

### 3.1. Error-Control Coding Leaks Information

First, let us consider the effect of adding an error-control code at Layer *L* in terms of $H(M|Z^{n})$ when the eavesdropper’s channel is a binary symmetric channel (BSC), and no layers of coding are used besides the one layer of error-control coding. The BSC flips bits at Eve’s receiver independently with probability *p*. Let us consider only a three bit transmission over this channel, where bits *A* and *B* are chosen independently at random, and bit

(12) C=A⊕B,

where ⊕ indicates addition in $F_{2}$. Bit *C* is then generated by one of the simplest parity-check equations possible for error control over bits *A* and *B*. Let the corresponding outputs from the channel for *A*, *B*, and *C*, be given as

(13) $$\begin{array}{cc} A^{\prime } & =A⊕E_{A}, \end{array}$$

(14) $$\begin{array}{cc} B^{\prime } & =B⊕E_{B}, \end{array}$$

(15) $$\begin{array}{cc} C^{\prime } & =C⊕E_{C}, \end{array}$$

respectively, where

(16) $$E_{X}=\left\{\begin{array}{c} 1,\mathrm{with}\;\mathrm{probability}p,\\ 0,\mathrm{with}\;\mathrm{probability}1-p, \end{array}\right.$$

for each of the three independent cases: X=A,B,C.

We wish to calculate $H(A|A^{\prime },B^{\prime },C^{\prime })$ and compare the quantity to $H\left(A|A^{\prime }\right)=H_{2}\left(p\right)$ to see the secrecy effect brought on by this simplest of codes, where $H_{2}\left(p\right)$ is the binary entropy function evaluated at *p*. First, note that

(17) $$H\left(A|A^{\prime },B^{\prime },C^{\prime }\right)=-\sum_{a,a^{\prime },b^{\prime },c^{\prime }\in \left\{0,1\right\}}p\left(a^{\prime },b^{\prime },c^{\prime }|a\right)p\left(a\right)\log_{2}\frac{p\left(a^{\prime },b^{\prime },c^{\prime }|a\right)p\left(a\right)}{p(a^{\prime },b^{\prime },c^{\prime })}.$$

It can be shown that

(18) $$p\left(a^{\prime },b^{\prime },c^{\prime }\right)=\left\{\begin{array}{cc} \frac{1}{4}{\left(1-p\right)}^{3}+\frac{3}{4}p^{2}\left(1-p\right), & \mathrm{if}a^{\prime }⊕b^{\prime }=c^{\prime },\\ \frac{3}{4}p{\left(1-p\right)}^{2}+\frac{1}{4}p^{3}, & \mathrm{if}a^{\prime }⊕b^{\prime }\neq c^{\prime }. \end{array}\right.$$

Note that $a^{\prime }⊕b^{\prime }=c^{\prime }$ when there are an even number of bit flips through the BSC since a⊕b=c at the transmitter, but $a^{\prime }⊕b^{\prime }\neq c^{\prime }$ when there are an odd number of bit flips through the BSC for the same reason. Similarly,

(19) $$p\left(a^{\prime },b^{\prime },c^{\prime }|a\right)=\left\{\begin{array}{cc} \frac{1}{2}p^{2}\left(1-p\right)+\frac{1}{2}{\left(1-p\right)}^{3}, & \mathrm{if}a^{\prime }⊕b^{\prime }=c^{\prime },a\;=a^{\prime },\\ p^{2}\left(1-p\right), & \mathrm{if}a^{\prime }⊕b^{\prime }=c^{\prime },a\;\neq a^{\prime },\\ p{\left(1-p\right)}^{2}, & \mathrm{if}a^{\prime }⊕b^{\prime }\neq c^{\prime },a\;=a^{\prime },\\ \frac{1}{2}p{\left(1-p\right)}^{2}+\frac{1}{2}p^{3}, & \mathrm{if}a^{\prime }⊕b^{\prime }\neq c^{\prime },a\;\neq a^{\prime }. \end{array}\right.$$

**Figure 3:**
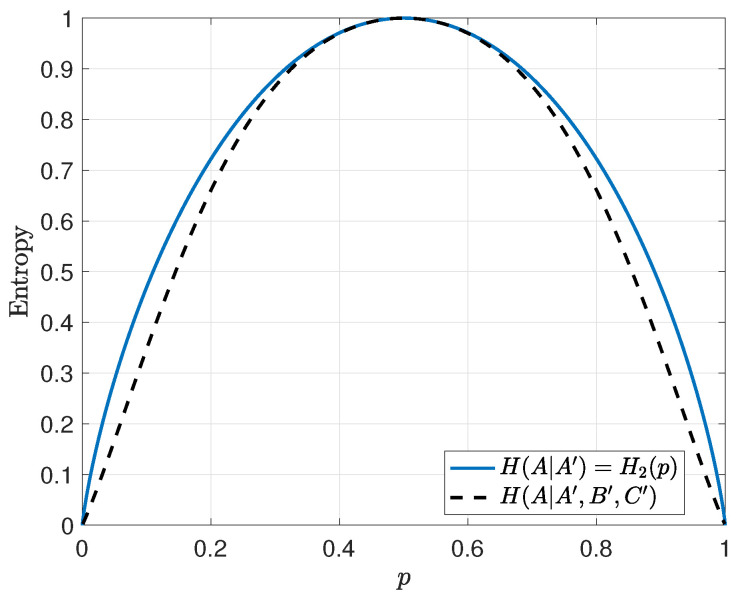
Conditional entropy of a message bit at the receiver for the uncoded and error-control coded case. Curves indicate that even a simple parity check leaks information compared to the uncoded case.

When we put this all together noting that $p\left(a\right)=p\left(a^{\prime }\right)=\frac{1}{2}$, then $H\left(A|A^{\prime },B^{\prime },C^{\prime }\right)<H\left(A|A^{\prime }\right)$ for all *p* except for the three trivial cases where p∈{0,0.5,1} as shown in Figure 3. The implication is that the introduction of a check sum that involves the bit *A* reduces the entropy of *A* at the receiver as long as the channel is not perfectly clean or perfectly noisy. This effect gets larger with the introduction of more checksums involving *A*.

### 3.2. Error-Control Coding Shapes Practical Secrecy

**Figure 4:**
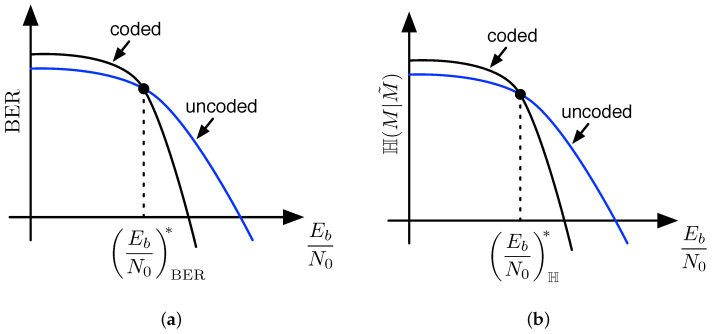
(**a**) illustration of the $\frac{E_{b}}{N_{0}}$ crossover point ${\left(\frac{E_{b}}{N_{0}}\right)}_{\mathrm{BER}}^{*}$ in bit-error rate curves for coded and uncoded data; (**b**) illustration of the $\frac{E_{b}}{N_{0}}$ crossover point ${\left(\frac{E_{b}}{N_{0}}\right)}_{H}^{*}$ in $H(M|\hat{M})$ curves for coded and uncoded data, whose existence implies that error-control coding increases practical secrecy below the crossover point and decreases practical secrecy above the crossover point.

It seems as if layers of error-control coding are doomed to leak information to the eavesdropper, and this is certainly true when secrecy is quantified using the traditional equivocation $H(M|Z^{n})$, as shown in the previous section. For the practical secrecy metric, however, $H(M|\tilde{M})$ does not necessarily indicate the leakage of information for some signal-to-noise ratios. Let us consider uncoded binary phase shift keying (BPSK) as compared to coded BPSK over a Gaussian channel, where the coding is a simple layer of error control coding as before. Figure 4a illustrates BER curves for the two cases, and points out that there exists an $\frac{E_{b}}{N_{0}}$ threshold value, which we call ${\left(\frac{E_{b}}{N_{0}}\right)}_{\mathrm{BER}}^{*}$, where the bit-error rates of the two cases are equivalent. For $\frac{E_{b}}{N_{0}}$ below this threshold, the coded case exhibits a higher error rate compared to the uncoded case, while for $\frac{E_{b}}{N_{0}}$ above this threshold, the coded case exhibits a lower error rate. Standard distance property arguments argue the existence of such a threshold for all possible error-control codes. Once the channel is degraded below a certain amount, errors in the channel are more likely to result in incorrect codewords at the output of the decoder than the correct codeword. What is not as obvious, although still somewhat intuitive, is that there also exists a threshold in $\frac{E_{b}}{N_{0}}$, which we call ${\left(\frac{E_{b}}{N_{0}}\right)}_{H}^{*}$ for the $H(M|\tilde{M})$ curve of a code, whereby $\frac{E_{b}}{N_{0}}$ below the threshold will result in a rise in practical secrecy and $\frac{E_{b}}{N_{0}}$ above the threshold will result in a decrease in practical secrecy. This idea is illustrated in Figure 4b.

It will be shown in Section 5 that the two threshold values ${\left(\frac{E_{b}}{N_{0}}\right)}_{\mathrm{BER}}^{*}$ and ${\left(\frac{E_{b}}{N_{0}}\right)}_{H}^{*}$ are not equal in many cases. Typically, we require worse signal-to-noise ratio at Eve’s receiver to operate left of ${\left(\frac{E_{b}}{N_{0}}\right)}_{H}^{*}$ than we do to operate to the left of ${\left(\frac{E_{b}}{N_{0}}\right)}_{\mathrm{BER}}^{*}$, which satisfies our intuition that practical secrecy based on $H(M|\tilde{M})$ should be easier to achieve than weak or strong secrecy based on $H(M|Z^{n})$, but harder to achieve than secrecy based only on bit-error rates. It is furthermore true that layers of error-control coding may be used to shape secrecy benefit curves B(C) for codes C at other layers. Examples of this idea are given in Section 5.

## 4. Layers of Coding for Secure Data Transfer

In this section, we consider several layers of coding that are meant to secure the message against eavesdropping, and analyze the utility of keyless scrambling (and interleaving as a special case), in addition to coset-based secrecy coding.

### 4.1. Practical Security Coding Layers

Simple rate-one layers of coding have been argued to fill practical roles of error propagation in numerous works including [13], and even some of our own past works [10,18,19]. While the BER may be affected by such layers of coding, we show in this section that $H(M|\tilde{M})$ is unaffected by rate one bijective coding layers.

Let *S* be a rate one encoder in the form of a square k×k binary matrix, where the 1×k output of the encoder $x_{\mathrm{out}}$ is produced from the 1×k input of the encoder $x_{\mathrm{in}}$ by the encoding operation

(20) $$x_{\mathrm{out}}=x_{\mathrm{in}}S.$$

Suppose that $S^{-1}$ is the decoder so that

(21) $$x_{\mathrm{in}}=x_{\mathrm{out}}S^{-1}$$

for all possible $x_{\mathrm{in}}$. Notice that *S* and $S^{-1}$ define a bijection between $F_{2}^{k}$ and itself, and constitute a layer of rate-one coding.

**Theorem** **1.** *A rate-one encoding function S that forms a bijection between $F_{2}^{k}$ and itself for any positive integer k, has secrecy benefit B(S)=0 over a discrete memoryless symmetric eavesdropper’s channel.*

**Proof.**  Recall that

(22) $$B\left(S\right)=H_{S}\left(M|\tilde{M}\right)-H_{U}\left(M|\tilde{M}\right),$$

where *M* is coded with *S* to produce *X*, which is transmitted over the eavesdropper’s channel to produce *Z*. Since the channel is symmetric, then its noise properties are independent of the choice of x∈X. *Z* is then mapped to $\tilde{M}$ via $S^{-1}$. Note the transition probabilities $p_{\tilde{M}|M}\left(\tilde{m}|m\right)$ for this encoding, and recognize that fixing m∈M produces |M| probabilities whose order is dependent on the choice of *m*, but whose collection of values are independent of the choice of *m*, as in (10). Since *S* and $S^{-1}$ define a bijection on $F_{2}^{k}$, these probabilities are identical to the collection of probabilities $p_{Z|X}\left(z|x\right)$ that define hard decisions over the eavesdropper’s channel. Thus,

(23) $$-\sum_{\tilde{m}\in M}p\left(\tilde{m}|m\right)\log_{2}p\left(\tilde{m}|m\right)=-\sum_{z\in Z}p\left(z|x\right)\log_{2}p\left(z|x\right),$$

which implies that

(24) $$H_{S}\left(M|\tilde{M}\right)=H_{U}\left(M|\tilde{M}\right),$$

by application of (10). □

The implications of this theorem are that keyless layers of scrambling and interleaving (which is a special case of scrambling) provide no secrecy benefit in terms of $H(M|\tilde{M})$. We give the following as a corollary to Theorem 1.

**Corollary** **1.** *Let M be encoded to produce X, which is transmitted over a discrete memoryless channel resulting in Z, which is decoded to produce $\tilde{M}$. If the encoding function S is a rate-one bijective function between $F_{2}^{k}$ and itself for any positive integer k, then*

(25) $$H\left(X|Z\right)=H\left(M|Z^{n}\right)=H\left(M|\tilde{M}\right).$$

*This indicates that the stricter form of the secrecy benefit calculated as $H\left(M|Z^{n}\right)-H_{U}\left(M|\tilde{M}\right)$ is also zero.*

**Proof.**  Two uses of the data processing theorem result in

(26) $$I\left(X;Z\right)\geq I\left(M;Z\right)\geq I\left(M;\tilde{M}\right),$$

which can be rewritten in terms of equivocations as

(27) $$H\left(X|Z\right)\leq H\left(M|Z\right)\leq H\left(M|\tilde{M}\right).$$

Thus, the corollary is proved by Theorem 1. □

Note that these results hold only for encoding functions *S* that form a bijection over $F_{2}^{k}$, which is not true if multiple coded blocks are interleaved or scrambled together. Thus, there may still be some benefit to inter-block rate-one encoding functions, although intra-block rate-one encoding functions appear to have no effect of increasing the information theoretic security.

### 4.2. Information Theoretic Security Coding Layers

In this section, we contrast the simple rate-one encoding schemes from the previous section with coset codes for the wiretap channel [5,27]. While it was shown that rate-one codes are of no effect over symmetric discrete memoryless channels (and hence, hard decision Gaussian channels), wiretap codes, on the other hand, are of immense effect in increasing the practical secrecy.

Let *G* be the (n−k)×n generator matrix of an (n,n−k) binary linear block code C. Then, let $G^{\prime }$ be a k×n matrix such that $G^{*}=\left[\begin{array}{c} G\\ G^{\prime } \end{array}\right]$ is a full rank n×n matrix in $F_{2}$. Let *H* be the k×n parity check matrix associated with C. Then, the encoding function of a coset wiretap code computes a codeword as

(28) $$x^{n}=\left[\begin{array}{cc} m^{\prime } & m \end{array}\right]G^{*},$$

where $m^{\prime }$ is a randomly chosen 1×(n−k) vector in $F_{2}^{n-k}$. Note that this encoding function picks a specific element of a coset of C as the codeword $x^{n}$, where *m* chooses the coset of C and $m^{\prime }$ chooses the codeword within the coset. Codes such as these were originally showcased in [5], and have been used in many wiretap code constructions [8]. Confusion about the message is achieved as an eavesdropper uncertainty of the proper coset is increased. Errors in the eavesdropper’s channel facilitate this confusion. Note that such a code has a secrecy rate of R=k/n, but the overhead of the code is used to cause confusion, rather than error correction. Since all codewords in the same coset have the same syndrome [25], codewords can be mapped back to messages via the calculation

(29) $$s=rH^{T},$$

where *r* is the received word at the receiver, and *s* is the syndrome of that word. Although it can be made so that m=s as in [4], storing the mapping from *s* to *m* is sufficient for decoding. These codes have been analyzed in great depth, and variants of them have been proposed to achieve weak, strong, and semantic secrecy [9] over various discrete memoryless wiretap channel models. In this work, we apply them as the Layer 1 code in Figure 2, and show how other layers combine to shape the secrecy benefit of these codes. We will also show in Section 5 that these codes are the only layers of this study with any significant help to achieving secure throughput.

## 5. Results and Discussion

In this section, we consider several different combinations of layers of coding, and show the BER curves, the practical secrecy $H(M|\tilde{M})$ curves, and the secrecy benefit B(C) curves. Here, C denotes the coding operation inclusive of all deployed layers. This set of results is presented for two different sets of parameters on the coding layers to showcase the general effect first using small algebraic codes and then that the trends hold for slightly larger codes. The following cases are all presented as combinations of coding layers for both sets of coding parameters:

- Zero layers of coding (uncoded case);
- One layer: error-control coding (ECC);
- One layer: scrambling;
- One layer: interleaving (special case of scrambling);
- One layer: coset coding;
- Two layers: scrambling, then ECC;
- Two layers: interleaving, then ECC;
- Two layers: coset coding, then ECC;
- Three layers: coset coding, then scrambling, then ECC.

BPSK modulation is applied to all Layer *L* encoding outputs.

### 5.1. Algebraic Code Examples with Inter-Block Processing

In this section, we use the (7,4) Hamming code. ECC layers use the Hamming code for error correction, while the coset coding layer is chosen to have the same rate of R=4/7. The dual of the Hamming code (the (7,3) simplex code) is used as the base linear code for the coset coding to accomplish this. Scramblers and interleavers are applied at the smallest block level; thus, *S* is a 4×4 binary matrix with inverse $S^{-1}$ in $F_{2}$. Although all analysis to this point has only allowed for block-by-block processing, we allow inter-block processing to match the dimensions of the chosen codes. This requires buffering between encoders and decoders in practice. All decoders are hard-decision in this section, and decoding the ECC layers is performed using the syndrome method [25]. We employ state-of-the-art decoders in the next section of examples.

**Figure 5:**
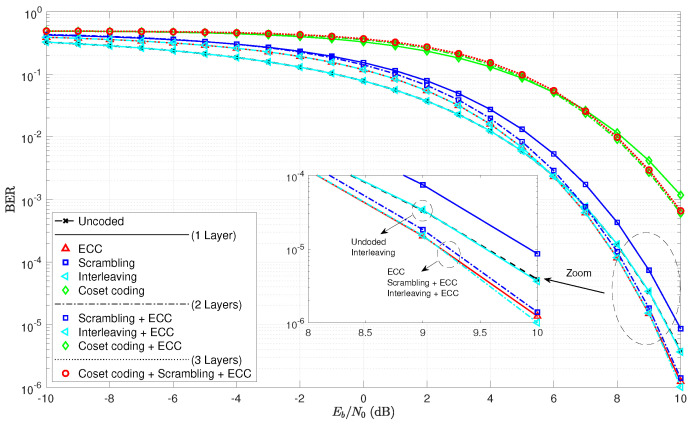
Bit-error rate curves for various combinations of coding layers and the set of algebraic code examples.

As a first step to the analysis, consider the BER curves in Figure 5. Note that the single layer of interleaving does not even affect the BER curve, as it lays directly over the uncoded curve. This is true since an error prior to deinterleaving remains only one error in the deinterleaver output, even though its location has changed. The ECC curves exhibit a crossover point ${\left(\frac{E_{b}}{N_{0}}\right)}_{\mathrm{BER}}^{*}$ around 6 dB. As expected, scrambling does appear to increase the BER, and the cases with wiretap codes in play have much higher error rates.

**Figure 6:**
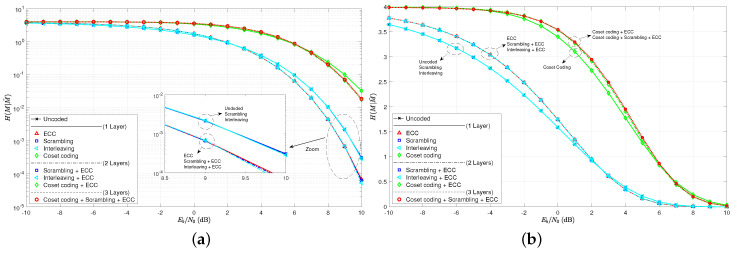
(**a**) practical secrecy $H(M|\tilde{M})$ curves as a function of $\frac{E_{b}}{N_{0}}$ on a log scale for the algebraic code examples; (**b**) practical secrecy $H(M|\tilde{M})$ curves as a function of $\frac{E_{b}}{N_{0}}$ on a linear scale for the algebraic code examples.

The practical secrecy curves for these same coding scenarios are given in Figure 6 on both a log scale and a linear scale. We see here much less difference between the several cases for $H(M|\tilde{M})$ than we saw for BER in Figure 5. When considering the BER, we had seven unique curves between the nine tested scenarios. Only interleaving appeared not to make a difference in the curves. Now, when considering $H(M|\tilde{M})$ curves, however, there appear to only be four unique curves. Scrambling and interleaving are both shown to be of no effect as predicted. The four unique cases are essentially the uncoded case, the ECC single layer case, the coset coding single layer case, and the coset coding plus ECC double layer case. We see the threshold ${\left(\frac{E_{b}}{N_{0}}\right)}_{H}^{*}$ at around 2.5 dB, which, as predicted is much less than ${\left(\frac{E_{b}}{N_{0}}\right)}_{\mathrm{BER}}^{*}$. This implies that practical secrecy is harder to achieve than secrecy based on BER, as discussed previously.

**Figure 7:**
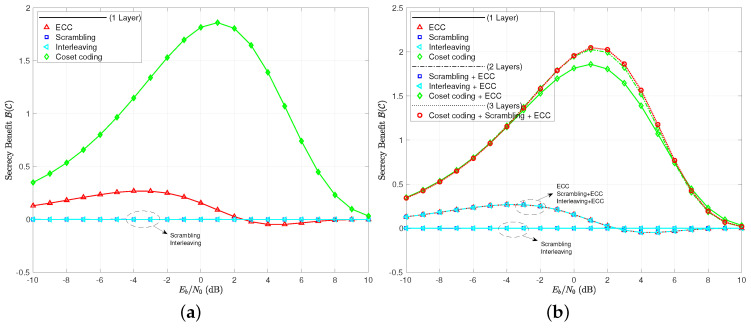
(**a**) secrecy benefit to coding B(C) for single layer codes; (**b**) secrecy benefit to coding B(C) for all coding scenarios tested.

Finally, we explore the secrecy benefit of the many cases in Figure 7. Of greatest interest is that even coset coding only provides a positive secrecy benefit when compared to the uncoded case for a range of $\frac{E_{b}}{N_{0}}$ around the usual operating points of radio receivers (roughly −10 dB to +10 dB). For lower $\frac{E_{b}}{N_{0}}$, the channel is so bad that the uncoded case is essentially secure on its own, while, for higher $\frac{E_{b}}{N_{0}}$, even the coset coding communicates the information error free. In either case, the code cannot be deemed to be helping the situation. The ECC layer has a positive secrecy benefit up until ${\left(\frac{E_{b}}{N_{0}}\right)}_{H}^{*}$, and a negative secrecy benefit afterwards. The secrecy benefits of scrambling and interleaving are shown to be zero as expected. When we consider multi-layer coding, it appears that more is going on. Scrambling appears to help, although we need to remember that we have allowed inter-block scrambling for this case. Essentially, the only significantly different secrecy benefit curve in the multi-layer coding schemes is the one that combines ECC with coset coding. We see hints of a shaping effect of B(C), where the ECC can be used to affect the secure and reliable regions of signal-to-noise ratio, even when codes are small.

### 5.2. Code Examples with Intra-Block Processing Only

In this section, we are more strict as to ensuring that each message block is processed independently. That is, the dimensions of Layer 2 and Layer 3 codes are set equal to the blocklengths of Layer 1 and Layer 2 codes, respectively. We also increase the dimension of the Layer 1 encoder from 4 in the last section to 15 in this section. While the dimension is still small, it is necessary to present high fidelity simulation results with the new metrics, and even the small size shows the benefit of each layer and how combinations of layers can work together to achieve specific effects. All ECC and coset coding layers are encoded at R=1/2. The parity-check matrix that governs ECC layers is chosen using the socket approach to irregular low-density parity-check (LDPC) code construction [28] with the edge-perspective degree distribution pair [25]

(30)λ(x)=0.3157x2+0.41672x3+0.4381x7,(31)ρ(x)=0.4381x6+0.5619x7.

This same code is used as the base linear code for the coset coding. Finally, soft-decision decoding is used whenever possible, although some layers require hard decisions to do the decoding operations, e.g., scrambling and coset decoding. Soft demodulation and soft-information belief propagation at the ECC decoder are both used.

**Figure 8:**
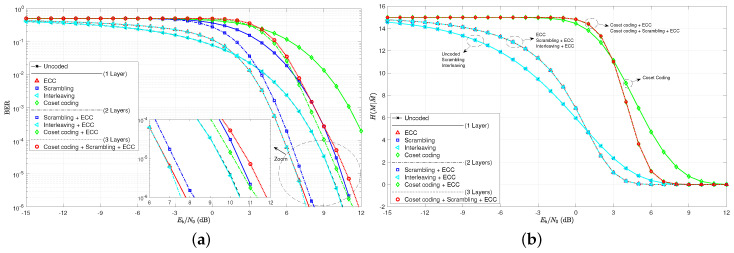
(**a**) bit-error rate curves for various combinations of coding layers and the set of irregular coding examples; (**b**) practical secrecy $H(M|\tilde{M})$ curves as a function of $\frac{E_{b}}{N_{0}}$ on a linear scale for the irregular code examples.

This new set of code parameters give very similar results as those given by the algebraic set of codes based on Hamming and simplex codes. Note in Figure 8 the BER and practical secrecy curves. Again, we see plenty of differences across the coding layer combinations for BER, but only four cases for $H(M|\tilde{M})$. Only ECC and coset codes make any difference in practical secrecy. The thresholds of crossover come in around ${\left(\frac{E_{b}}{N_{0}}\right)}_{\mathrm{BER}}^{*}\approx 2$ dB and ${\left(\frac{E_{b}}{N_{0}}\right)}_{H}^{*}\approx 1$ dB, again indicating that it is harder to make a code help the secrecy cause when using $H(M|\tilde{M})$ as the measuring stick, rather than BER.

**Figure 9:**
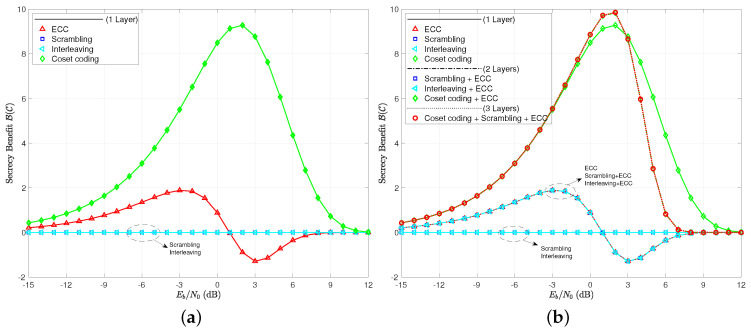
(**a**) secrecy benefit to coding B(C) for single layer codes; (**b**) secrecy benefit to coding B(C) for all coding scenarios tested.

The secrecy benefit curves are given in Figure 9, where we see again that scrambling and interleaving are of no effect when measuring practical secrecy. The notion of using a layer of ECC to shape the secrecy benefit of a coset code is a bit more believable when looking at the multi-layer coding schemes here. Note that the combination of these two layers increases the peak secrecy benefit, moves its location slightly to the left, and brings about a reliable region of operating signal-to-noise ratios sooner than the coset coding only case. Scrambling and interleaving, once again, prove to be useless in increasing the practical secrecy or secrecy benefit in any way.

## 6. Conclusions

In conclusion, this paper provides a fresh approach to information theoretic security over the Gaussian wiretap channel for finite blocklength codes. It is shown how to quantify the practical secrecy and the secrecy benefit of a code, and this approach is used to systematically analyze the effects of various combinations of layers of coding in a concatenated coding scheme. Scrambling and interleaving are shown to have no effect on the practical secrecy, and thereby achieve zero secrecy benefit. On the other hand, error-control coding and wiretap coding are shown to have a significant secrecy benefit over uncoded transmissions. Layers of error-control coding may be used to shape the secrecy benefit of wiretap coding when concatenated coding schemes are employed.

## Abbreviations

The following abbreviations are used in this manuscript:

BPSK	binary phase shift keying
BER	bit-error rate
BSC	binary symmetric channel
LDPC	low-density parity-check
